# IL-4Rα blockade reduces influenza-associated morbidity in a murine model of allergic asthma

**DOI:** 10.1186/s12931-021-01669-0

**Published:** 2021-03-02

**Authors:** Kimia Shahangian, David A. Ngan, H. H. Rachel Chen, Yeni Oh, Anthony Tam, Jing Wen, Chung Cheung, Darryl A. Knight, Delbert R. Dorscheid, Tillie L. Hackett, Michael R. Hughes, Kelly M. McNagny, Jeremy A. Hirota, Masahiro Niikura, S. F. Paul Man, Don D. Sin

**Affiliations:** 1 Centre for Heart Lung Innovation, St. Paul’s Hospital, University of British Columbia, Room 166, 1081 Burrard Street, Vancouver, BC V6Z 1Y6 Canada; 2 Department of Medicine, University of British Columbia, Vancouver, BC Canada; 3 School of Biomedical Sciences and Pharmacy, University of Newcastle, Callaghan, Australia; 4 The Biomedical Research Centre, University of British Columbia, Vancouver, BC Canada; 5 Division of Respirology, Department of Medicine, McMaster University, Hamilton, ON Canada; 6 Department of Health Sciences, Simon Fraser University, Vancouver, BC Canada

## Abstract

**Background:**

Asthma was identified as the most common comorbidity in hospitalized patients during the 2009 H1N1 influenza pandemic. We determined using a murine model of allergic asthma whether these mice experienced increased morbidity from pandemic H1N1 (pH1N1) viral infection and whether blockade of interleukin-4 receptor α (IL-4Rα), a critical mediator of T_h_2 signalling, improved their outcomes.

**Methods:**

Male BALB/c mice were intranasally sensitized with house dust mite antigen (Der p 1) for 2 weeks; the mice were then inoculated intranasally with a single dose of pandemic H1N1 (pH1N1). The mice were administered intraperitoneally anti-IL-4Rα through either a prophylactic or a therapeutic treatment strategy.

**Results:**

Infection with pH1N1 of mice sensitized to house dust mite (HDM) led to a 24% loss in weight by day 7 of infection (versus 14% in non-sensitized mice; p < .05). This was accompanied by increased viral load in the airways and a dampened anti-viral host responses to the infection. Treatment of HDM sensitized mice with a monoclonal antibody against IL-4Rα prior to or following pH1N1 infection prevented the excess weight loss, reduced the viral load in the lungs and ameliorated airway eosinophilia and systemic inflammation related to the pH1N1 infection.

**Conclusion:**

Together, these data implicate allergic asthma as a significant risk factor for H1N1-related morbidity and reveal a potential therapeutic role for IL-4Rα signalling blockade in reducing the severity of influenza infection in those with allergic airway disease.

## Introduction

The 2009 H1N1 influenza A pandemic strain (pH1N1, A(H1N1)pdm09) caused 18,500 deaths, over 12,500 of which were reported in the United States [1, 2]. However, studies have estimated the true impact of the pandemic to lie between 151,000–575,000 deaths worldwide [2]. Generated through triple reassortment, the severity of this strain was due, in part, to a lack of pre-existing immunity in the population [3]. Asthma, the most common risk factor for severe cases, was identified in 17–27% of hospitalized patients [4, 5]. With over 300 million people suffering from asthma worldwide, urgent action is needed to protect this population from highly virulent influenza A strains [6, 7].

Asthma is a complex disease that is associated with airway inflammation, remodeling, and hyper-reactivity. Allergic asthma, the most common endotype, is characterized by a predominant T_h_2-skewed phenotype, with patients exhibiting eosinophilia and elevated levels of interleukin (IL)-4, IL-5, and IL-13 in the airways and lung tissue [8–12]. IL-4 and IL-13 are thought to play an important role in asthma pathogenesis by inducing eotaxin (an eosinophil chemoattractant), increasing mucin glycoproteins (and causing mucous hyperplasia), and augmenting IgE-related hypersensitivity reactions [13–15]. T_h_2 cytokines have also been implicated in promoting viral exacerbations. For example, in an experimental rhinovirus infection model, patients with asthma were shown to develop a greater severity of lower respiratory tract symptoms and impaired lung function, which was associated with increased expression of IL-4, IL-5, and IL-13 in the bronchoalveolar lavage fluid (BALF) [16]. Allergic asthma models have also demonstrated that IL-33, an upstream T_h_2 cytokine, may enhance influenza-induced exacerbations by dampening the expression of interferon (IFN)-β, a key antiviral mediator [17].

As the common receptor for both IL-4 and IL-13 signaling, blockade of IL-4 receptor alpha (IL-4Rα) has been touted as a method of alleviating asthma symptoms and severity [18]. Murine asthma models have demonstrated a significant reduction in airway hyperresponsiveness, lung eosinophilia, and goblet cell metaplasia upon monoclonal antibody (mAb) blockade of IL-4Rα [19]. Clinical trials using a humanized mAb targeting IL-4Rα have demonstrated up to 87% reduction in exacerbation rates and a 320 mL increase in FEV_1_ in patients with moderate to severe asthma [20, 21]. While the blockade of IL-4Rα has proven to be effective in reducing asthma severity, its clinical potential in ameliorating exacerbations of highly virulent influenza A infections has yet to be investigated. Here, we evaluated this using a murine model in which mice were sensitized to an extract from a common aeroallergen house dust mite (HDM) and intranasally infected with pH1N1. We found that HDM-sensitized mice had greater pH1N1-related morbidity which was alleviated by IL-4Rα mAb blockade. Our in vivo murine model results highlight the therapeutic potential of IL-4Rα-mediated therapies in asthmatic patients during influenza pandemics.

## Methods

Detailed methods are provided in the Methods section in Additional file 1.

### Mice

Male BALB/c mice (6–8 weeks old) were obtained from Jackson Laboratory (Bar Harbour, ME). Mice were housed in a specific pathogen-free environment with protocols approved by the Animal Care and Biosafety Committees of the University of British Columbia.

### Acute house dust mite sensitization and pandemic H1N1 infection model

**Fig. 1:**
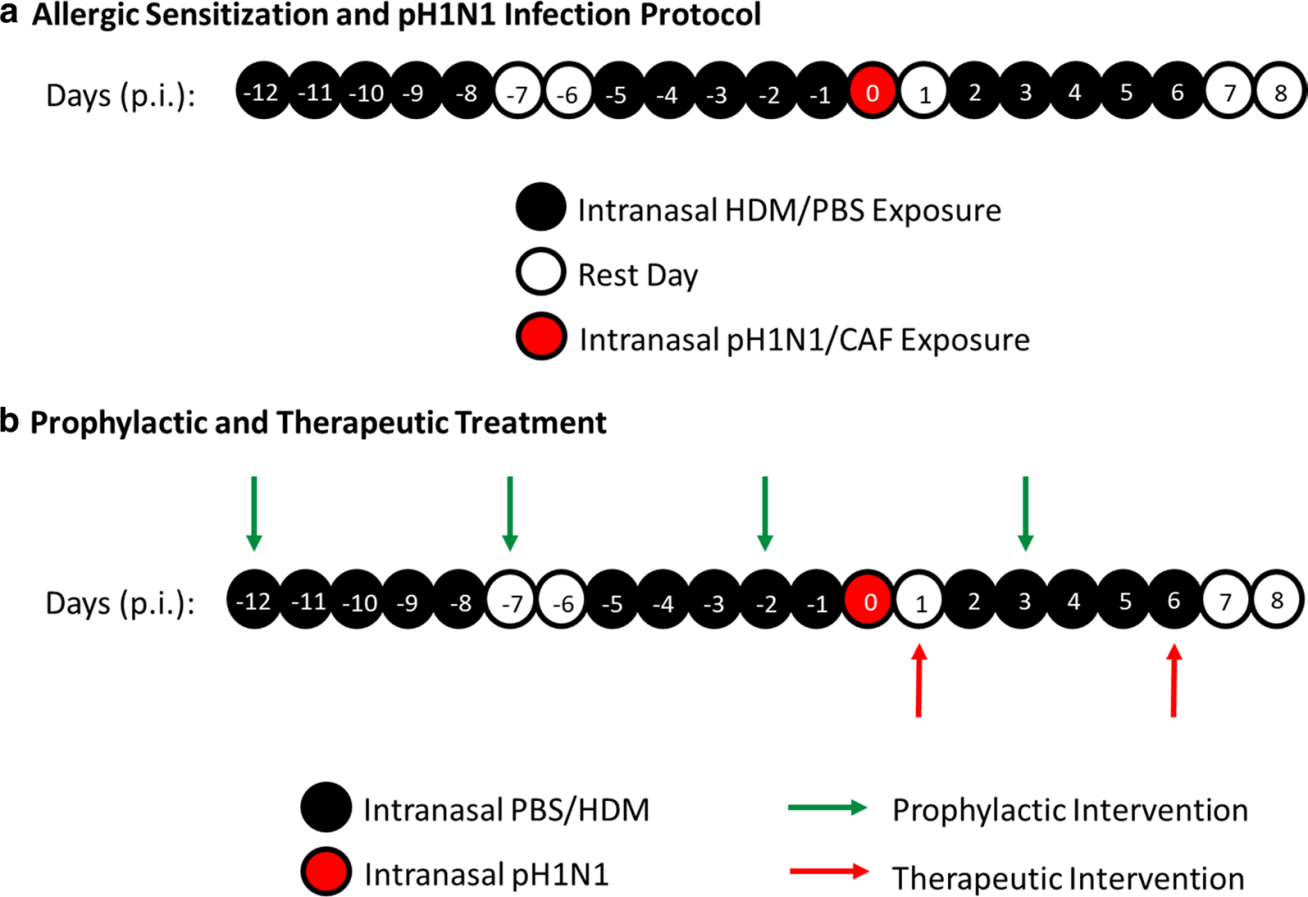
Experimental design. **a** To create an allergic sensitization model, mice were intranasally exposed to either phosphate-buffered saline (PBS) or house dust mite (HDM) extract, 5 days per week for 3 consecutive weeks. A single intranasal dose of pandemic H1N1 (pH1N1) was administered on day 0. Control animals instead received chorioallantoic fluid (CAF) from non-infected eggs as a vehicle control (n = 10 per group, except n = 9 for HDM + pH1N1). **b** Using the allergic sensitization and pH1N1 infection protocol, HDM-sensitized mice were intraperitoneally administered anti-IL-4Rα or IgG1 isotype control monoclonal antibodies through either a prophylactic (on days-12, -7, -2, 1, and 6 post-infection (p.i.)) or a therapeutic treatment strategy (on days 1 and 6 p.i.). Control animals were intranasally exposed to PBS or HDM, and received intraperitoneal administrations of the vehicle control, PBS

To create an allergic asthma model, mice were intranasally sensitized with 25 µg of HDM (Der p 1) delivered in 35 µL of phosphate-buffered saline (PBS). Control mice received PBS vehicle alone. Intranasal PBS or HDM instillations were performed 5 days per week for two consecutive weeks, followed by infection with pH1N1 (50 µL, 10^6.4^ EID_50_/mL in chorioallantoic fluid (CAF)) or CAF) from non-infected eggs (vehicle control). Intranasal PBS or HDM instillations were continued for another week. Mice were sacrificed on day 8 post-pH1N1 infection (n = 10 per group, except n = 9 for HDM + pH1N1) or through two time-course studies (mice sacrificed either on days 0, 2, 4, 6, and 8 post-infection, or on days 0, 1, 3, 5, and 8 post-infection, n = 5 per group per day). Additional protocol details can be found in Fig. 1 and the Methods section in Additional file 1.

### Prophylactic and therapeutic intervention designs

pH1N1-infected mice were intranasally exposed to PBS or HDM and received 2 mg of either anti-IL-4Rα (4-3) or IgG1 isotype control (4G8) mAb (Amgen, Seattle, WA) through an intraperitoneal route, as previously described [19, 22]. Anti-IL-4Rα immunotherapy was administered through either a prophylactic (on days 2, 7, and 12 days prior to and 3 days following pH1N1 infection) or a therapeutic strategy (on days 1 and 6 following pH1N1 infection), as outlined in Fig. 1 and the Methods section in Additional file 1.

### Quantitative polymerase chain reaction (qPCR)

RNA was extracted from homogenized lung tissue. To quantify viral load, qPCR was performed on triplicates of RNA samples and viral standards ranging from 10^3^ to 10^9^ RNA copies. The expression of interferon stimulated genes (ISGs, i.e. OAS1, RIG-I, MX-1, IFITM3, ISG-15, and Viperin) were quantified using qPCR relative to the expression of the control gene GAPDH, and analyzed using the comparative Ct method (ΔΔCt) [23]. See Additional details in the Methods section in Additional file 1.

### Lung histopathology and morphometry

The left lung was inflated with 400 µL of 10% formalin and fixed for 24 h. Paraffin-embedded sections (3 µm) were stained with periodic acid-Schiff (PAS), and analyzed by applying a colour segmentation algorithm using Aperio ImageScope software (Leica Biosystems, Wetzlar, Germany). The basal border of the epithelium was traced, and the level of staining is expressed as the number of positive and strong positive pixels per µm of basement membrane. Airway epithelial thickness was measured in FFPE-mouse lung tissues and stained with PAS. Airway epithelial thickness was determined by normalizing the area enclosed between the apical surface and the basement membrane (BM) to the length of the BM [24]. Sub-epithelial wall thickness was determined by normalizing the area enclosed between the BM and the adventitia to the length of the BM. All measurements were quantified using the Aperio ScanScope software. The histopathological assessment of tertiary lymphoid structures was performed as previously described [25]. The fractional lymphoid tissue area was quantified on PAS-stained slides and normalized to total lung cross-sectional area and expressed as a percentage [25]. Additional details on tissue sectioning can be found in the Methods section in Additional file 1.

### Bronchoalveolar lavage fluid and plasma analysis

IL-4, IL-5, IFN-γ, eotaxin, IL-6, and IP-10 concentrations were measured by a multiplex assay, while IL-33 and IFN-β concentrations were determined by ELISA. Additional details on sample collection and processing can be found in the Methods section in Additional file 1.

### Statistical analysis

D’Agostino-Pearson test was performed to determine normality prior to analysis. Weight was analyzed using a two-way ANOVA with a Bonferroni correction, while protein and gene expression data were analyzed using a two-way ANOVA with Sidak’s multiple comparisons test and the Mann–Whitney U test. All other data were analyzed using a two-tailed Student’s t-test (GraphPad Prism version 5.0, La Jolla, CA). Data are expressed as mean ± SEM, and P values < 0.05 were considered significant.

## Results

### HDM-sensitized mice experience excessive weight loss following pH1N1 infection

**Fig. 2:**
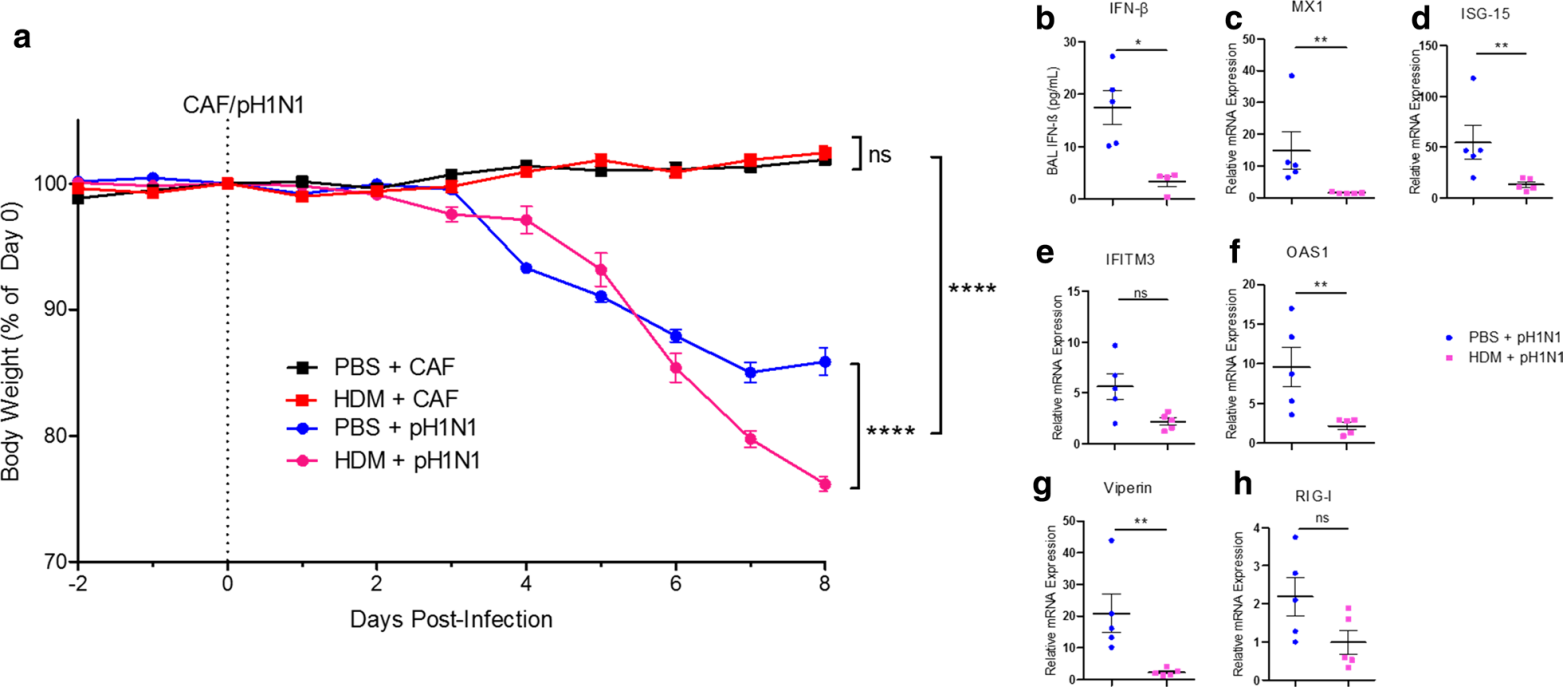
House dust mite (HDM)-sensitization induces greater pandemic H1N1 (pH1N1)-associated weight loss and a dampened innate antiviral response. Mice were intranasally exposed to phosphate-buffered saline (PBS) or HDM for three consecutive weeks, and a single intranasal inoculation of chorioallantoic fluid (CAF) or pH1N1 was performed on day 0. **a** Mice were weighed daily, and mean change in body mass is plotted as a percentage of weight on day 0. Data represent two independent experiments, and were compared using two-way ANOVA with a Bonferroni correction (n = 10 per group, except n = 9 for HDM + pH1N1). **b** ELISA was performed on the supernatant of bronchoalveolar lavage fluid (BALF) collected on day 4 post-infection to determine the protein concentration of IFN-β. **c**–**j** qPCR was performed on RNA from homogenized lung to determine the gene expression of Interferon stimulated genes (n = 5 per group). Data were compared using a Mann–Whitney U test, and are expressed as mean ± SEM. *P < 0.05; **P < 0.01; ****P < 0.0001; ns: not significant

To determine how underlying allergic sensitization and airway inflammation impact pH1N1-induced morbidity, mice were intranasally exposed to repeated doses of PBS or HDM, prior to and following intranasal inoculation with either pH1N1 or CAF (vehicle control). Weight loss was monitored daily as an indicator of disease severity. In CAF-exposed mice, HDM-sensitization alone failed to induce weight loss (Fig. 2). In contrast, pH1N1 infection induced significant (14.1% ± 3.4%) weight loss, which was exacerbated in HDM-sensitized mice (23.8% ± 1.7% weight loss) 7–8 days post-infection (Fig. 2a). HDM-sensitized mice also demonstrated higher immune cell infiltration into the airways, increased number of lymphoid structures and a higher concentration of total protein levels in lung homogenate supernatants on day 8 post-infection (Additional file 1: Fig. E7). Viral load was measured in homogenized lung tissue through a time-course experiment. HDM-sensitized mice exhibited higher viral titers beginning on day 4 and reaching over threefold higher levels by 8 days post-infection (see Additional file 1: Fig. E2A). The expression levels of interferon (IFN)-β and downstream interferon stimulated genes (ISGs) were also measured to assess host’s antiviral immune response. In addition to an elevated viral load, HDM-sensitized mice exhibited a fivefold reduction in the protein concentration of IFN-β in the airways (17.5 ± 7.2 pg/mL, vs. 3.4 ± 2.0 pg/ml in PBS-exposed mice, Fig. 2b). Furthermore, a dampened induction of several downstream ISGs, including MX1, OAS1, ISG-15, and viperin was observed on day 4 post-infection (Fig. 2d–j). See Additional file 1: Figs E2 and E3 for a complete time-course data of IFN-β and ISGs. We conclude that underlying allergic sensitization and airway inflammation exacerbate pathology associated with influenza virus infection.

### HDM-sensitization induces greater T_h_2 cytokine production in the airways

**Fig. 3:**
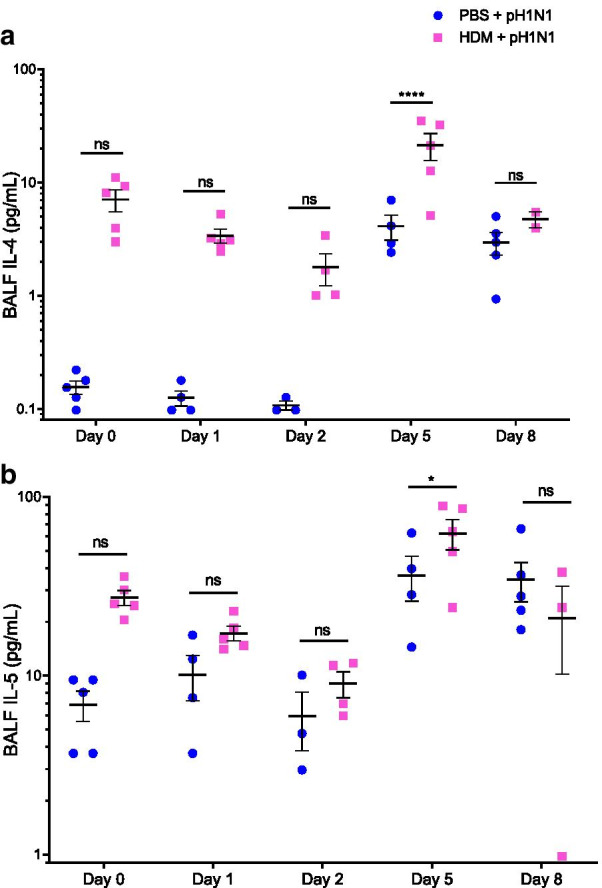
House dust mite (HDM)-sensitized and pandemic H1N1 (pH1N1)-infected mice demonstrate elevated T_h_2 cytokine levels in bronchoalveolar lavage fluid (BALF). BALF was obtained from pH1N1-infected mice on days 0, 1, 2, 5, and 8 post-infection (n = 5 per group per day). **a** IL-4 and **b** IL-5 protein levels were measured by a multiplex assay. Data were compared using a two-way ANOVA with Sidak’s multiple comparisons test, and are expressed as mean ± SEM. *P < 0.05 after correction for multiple comparisons; **P  < 0.01 after correction for multiple comparisons; ns: not significant

T_h_1 and T_h_2 inflammatory mediators were measured through a time-course experiment to profile the HDM-induced airway inflammation. Analysis of the bronchoalveolar lavage fluid (BALF) revealed elevated levels of IL-4 on days 0, 1, 2, and 5 (Fig. 3a), and IL-5 on days 0 and 5 post-infection (Fig. 3b). IL-33, a cytokine upstream of IL-5 and IL-13 production, was measured as an early inducer of the T_h_2 response. IL-33 protein levels were significantly elevated in the lung tissue of HDM-sensitized mice on days 0, 2, and 8 post-infection (see Additional file 1: Fig. E4A). In contrast, the BALF levels of IFN-γ, a T_h_1 inflammatory mediator, were significantly lower in HDM sensitized mice on day 5 post-infection (see Additional file 1: Fig. E4B). We conclude that HDM-sensitization leads to a T_h_2 skewed immune response and a dampening of T_h_1 responses to influenza.

### Prophylactic IL-4Rα blockade attenuates HDM-induced BALF eosinophilia

**Fig. 4:**
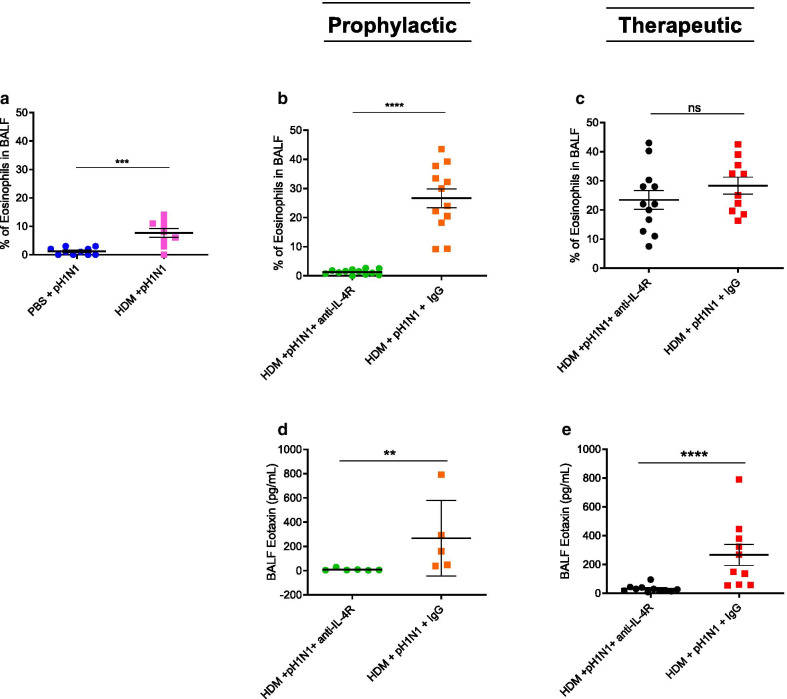
Systemic blockade of interleukin-4 receptor alpha (IL-4Rα) reduces house dust mite (HDM)-induced eosinophil recruitment to the airways. Bronchoalveolar lavage fluid (BALF) was obtained on day 8 post-infection from pandemic H1N1 (pH1N1)-infected mice. **a**–**c** The percentage of eosinophils was determined in a total of 200 counted cells, and was compared using two-tailed Student’s t-test. **d**–**e** Eotaxin levels in BALF supernatant were measured by a multiplex assay, and compared using the Mann–Whitney U test. Data are expressed as mean ± SEM. Blue/pink: intranasal phosphate-buffered saline (PBS) (n = 10) / intranasal HDM (n = 9); green/orange: intranasal HDM followed by prophylactic strategy of IL-4Rα blockade (n = 12) / IgG (n = 12); black/red: intranasal HDM followed by therapeutic strategy of IL-4Rα blockade (n = 12) / IgG (n = 10). **P < 0.01; ***P < 0.001; ****P < 0.0001; ns: not significant

BALF inflammatory infiltrates and differential cell counts were evaluated from day 8 post-infection. HDM-sensitization induced a significant increase in BALF eosinophils (7.67% ± 4.29%, vs. 1.2% ± 1.2% in PBS-exposed mice, Fig. 4a). As expected, prophylactic treatment with a mAb targeting IL-4Rα signalling after the induction of HDM allergic airway inflammation attenuated this response, resulting in a 21.5-fold reduction in BALF eosinophils (1.24% ± 0.78% eosinophils, Fig. 4b and c). In addition, we found that both prophylactic and therapeutic treatment courses led to a 28.4-fold and an 8.6-fold reduction, respectively, in BALF levels of eotaxin, an early marker of eosinophil recruitment (Fig. 4d, e). Corresponding to an increased percentage of eosinophils, HDM-sensitization was associated with a reduction in the percentage of macrophages, which was rescued upon IL-4Rα mAb blockade through a prophylactic treatment (see Additional file 1: Figure E5G–I). No differences were observed in the percentage of neutrophils or lymphocytes (see Additional file 1: Figure E5A–F).

### Prophylactic IL-4Rα blockade attenuates HDM-induced goblet cell metaplasia

**Fig. 5:**
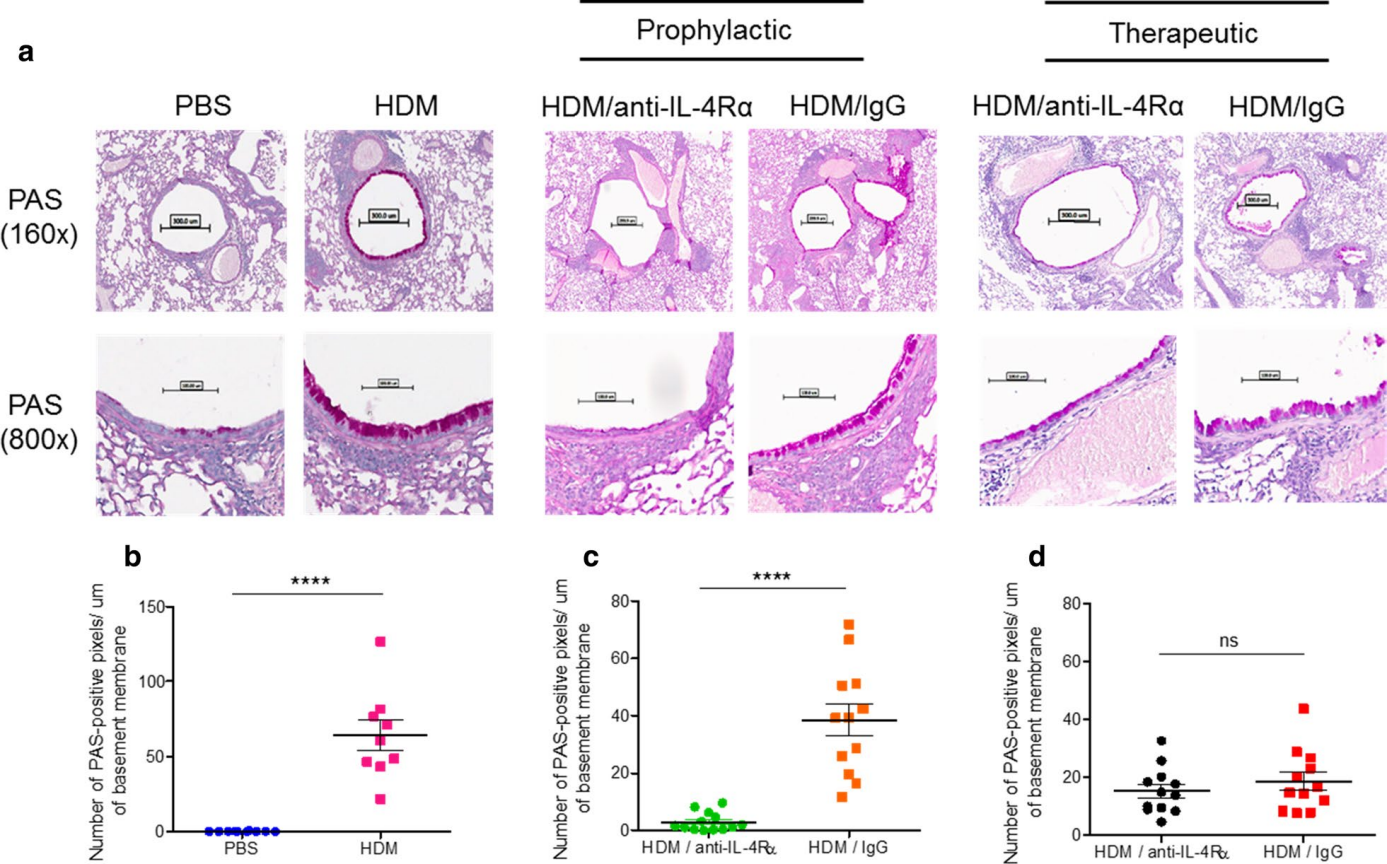
Systemic blockade of interleukin-4 receptor alpha (IL-4Rα) through a prophylactic strategy attenuates house dust mite (HDM)-induced goblet cell metaplasia. Lung tissue, obtained on day 8 post-infection from pH1N1-infected mice, was paraffin-embedded and stained with Periodic Acid Schiff (PAS). **a** Representative images were obtained at a magnification of 160× and 800×. **b**–**d** A colour segmentation algorithm was used to quantify positively stained cells. Data are expressed as mean ± SEM by two-tailed Student’s t-test. Blue/pink bars: intranasal phosphate-buffered saline (PBS) (n = 10) / intranasal HDM (n = 9); green/orange bars: intranasal HDM followed by prophylactic strategy of IL-4Rα blockade (n = 13) / IgG (n = 12); black/red bars: intranasal HDM followed by therapeutic strategy of IL-4Rα blockade (n = 12) / IgG (n = 12). **** P < 0.0001; ns: not significant

Goblet cells were quantified through their distinct morphology on PAS stained histologic sections in order to assess the extent of goblet cell metaplasia induced by HDM sensitization. HDM-sensitized mice demonstrated increased epithelial and airway wall thickness compared to PBS-exposed mice in both pH1N1-infected and non-infected mice (Additional file 1: Figure E8). Goblet cell frequency was normalized to the length of the basal membrane to account for differences in airway size. HDM-sensitized mice displayed extensive PAS staining representative of goblet cell metaplasia (64.1 ± 30.0 PAS^+^ pixels/µm of basement membrane, Fig. 5b), which was attenuated by prophylactic IL-4Rα mAb blockade (3.0 ± 3.2 PAS^+^ pixels/µm of basement membrane). However, we failed to see a complete attenuation when anti-IL-4Rα mAb was administered after the induction of allergic airway inflammation (15.3 ± 8.1 PAS^+^ pixels/µm of basement membrane, Fig. 5c and d). We conclude that prophylactic IL-4Rα blockade is sufficient to ameliorate the major hallmarks of allergic airway inflammation.

### IL-4Rα blockade reduces pH1N1 infection-induced weight loss in HDM-sensitized mice

**Fig. 6:**
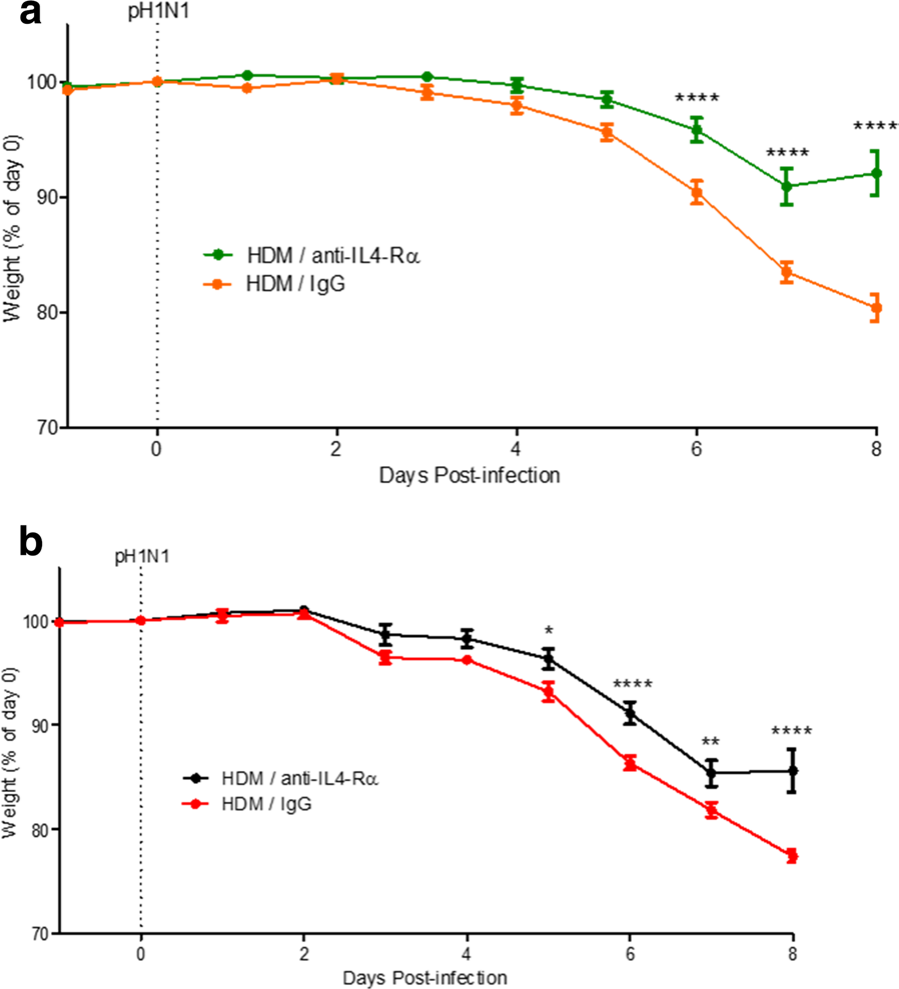
Systemic blockade of interleukin-4 receptor alpha (IL-4Rα) reduces house dust mite (HDM)-mediated excessive weight loss following pandemic H1N1 (pH1N1) infection. Mice were intranasally exposed to HDM for three consecutive weeks, and a single intranasal inoculation of pH1N1 was performed on day 0. Mean change in body mass is plotted as a percentage of weight on day 0 for **a** mice receiving IL-4Rα blockade (n = 13) or IgG (n = 12) through a prophylactic strategy (pooled from four independent experiments) and **b** mice receiving IL-4Rα blockade (n = 12) or IgG (n = 12) through a therapeutic strategy (pooled from two independent experiments). Data are expressed as mean ± SEM, and were compared using a two-way ANOVA with a Bonferroni correction. *P < 0.05, **P < 0.01, ****P < 0.0001

To determine the role of IL-4Rα signalling in HDM-induced morbidity following pH1N1 infection, weight loss was monitored in IgG control mice and those receiving either a prophylactic or a therapeutic treatment of IL-4Rα mAb blockade. pH1N1-induced weight loss was attenuated by both the prophylactic (7.9 ± 6.9%, vs. 19.6 ± 4% in IgG control mice) and the therapeutic (14.3 ± 7.2%, vs. 22.6 ± 2% in IgG control mice) treatment strategies in HDM-sensitized mice on days 6–8 post-infection (Fig. 6a and b).

### IL-4Rα blockade reduces viral load and pro-inflammatory cytokine concentration

**Fig. 7:**
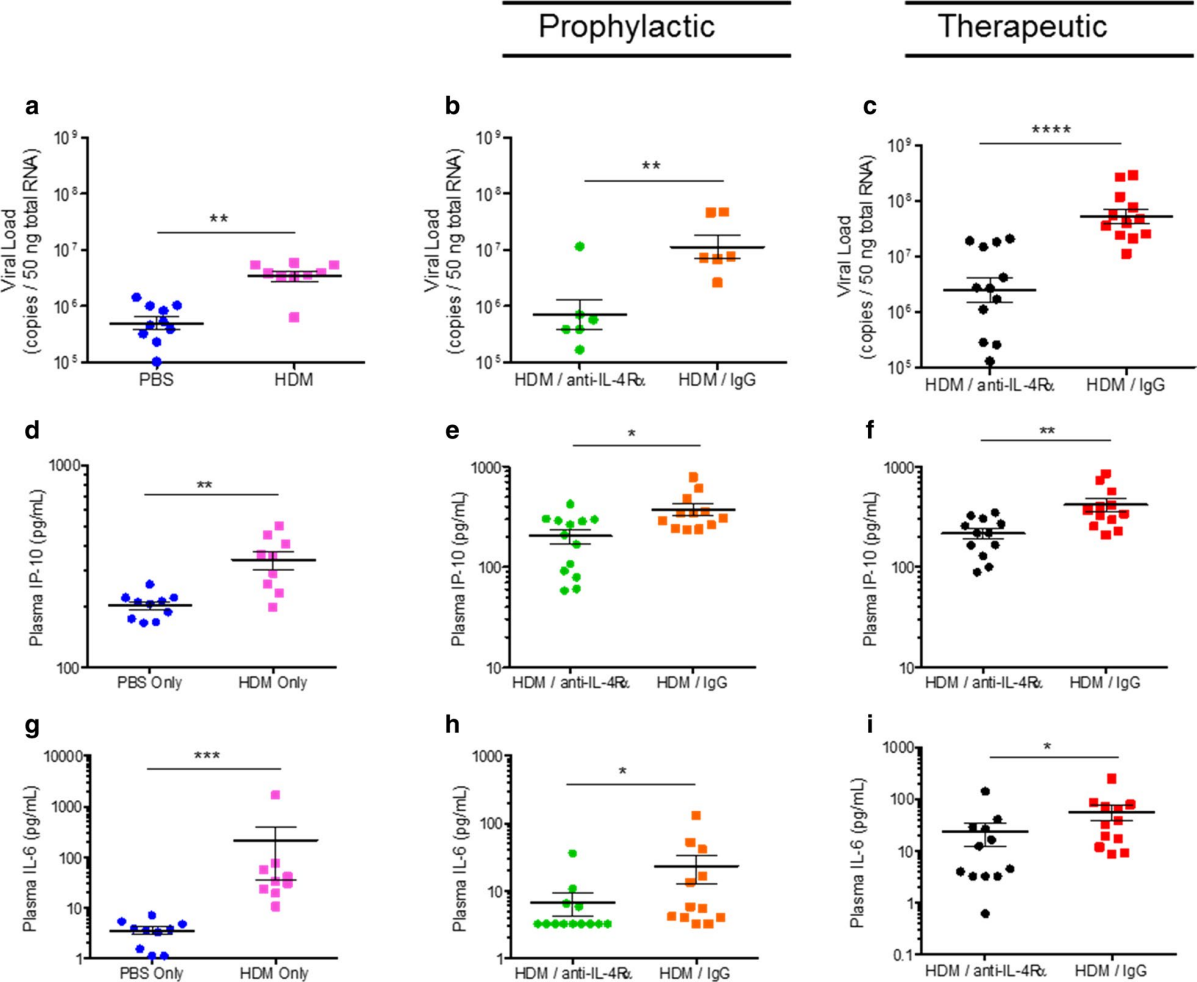
Systemic blockade of interleukin-4 receptor alpha (IL-4Rα) reduces house dust mite (HDM)-mediated excessive viral load and a systemic cytokine storm following pandemic H1N1 (pH1N1) infection. Lung tissue and plasma were obtained on day 8 post-infection from pH1N1-infected mice **a**–**c** qPCR was performed on RNA from homogenized lung tissue to measure the number of viral copies (pooled from two independent experiments), and results were compared using two-tailed Student’s t-test. Plasma was used to measure protein levels of **d**–**f** IL-6 and **g**–**i** IP-10. Data were compared using the Mann–Whitney U test, and are expressed as mean ± SEM. Blue/pink: intranasal phosphate-buffered saline (PBS) (n = 10) / intranasal HDM (n = 9); green/orange: intranasal HDM followed by prophylactic strategy of IL-4Rα blockade (n = 13) / IgG (n = 12); black/red: intranasal HDM followed by therapeutic strategy of IL-4Rα blockade (n = 12) / IgG (n = 12). *P < 0.05; **P < 0.01; ***P < 0.001; ****P < 0.0001; ns: not significant

To further explore the influence of IL-4Rα blockade on pH1NI-infected mice, viral loads were evaluated in the lungs of infected mice in the various treatment groups. Mice were sacrificed on day 8 post-infection, and qPCR was performed on homogenized lung tissue. HDM-sensitized mice demonstrated elevated copy numbers of the virus (10^6.5^ ± 10^0.3^ copies) compared with PBS exposed mice (10^5.7^ ± 10^0.3^ copies) (Fig. 7a). HDM-sensitized mice also demonstrated elevated levels of the pro-inflammatory cytokines IL-6 (Fig. 7d) and IFNγ-induced protein 10 (IP-10) (Fig. 7g) in plasma, but no differences were found in BALF (see Additional file 1: Figure E6A, D).

Administration of a mAb that blocks IL-4Rα signalling in HDM-sensitized mice induced a reduction in viral titres in both the prophylactic (10^5.8^ ± 10^0.6^ copies, vs. 10^7.1^ ± 10^0.5^ copies in IgG control mice) and therapeutic (10^6.4^ ± 10^0.8^ copies, vs. 10^7.7^ ± 10^0.4^ copies in IgG control mice) treatment groups (Fig. 7b, c). In addition, we observed a reduction in IL-6 (Fig. 7e–f) and IP-10 (Fig. 7h–i) protein levels in plasma and BALF (see Additional file 1: Figure E6B, C, E, F). We conclude that IL-4Rα mAb blockade reduces excessive viral burden associated with HDM-sensitization and the concentration of pro-inflammatory markers associated with asthma exacerbation.

## Discussion

Although the 2009 H1N1 pandemic affected a heterogeneous group of individuals, asthma was the most common risk factor leading to hospitalization and death [4, 5]. With over 300 million people worldwide suffering from asthma, new therapies are urgently needed to protect this vulnerable population from highly virulent influenza A infections [6]. Using a murine model of allergic airway inflammation, we showed that prior HDM-sensitization results in greater pH1N1-induced morbidity (as measured by weight loss). Importantly, we demonstrated that prophylactic or therapeutic use of a mAb that blocks IL-4Rα signalling, a clinically approved intervention strategy for moderate to severe asthma, can reduce the severity of inflammation and pathology related to pH1N1 infection [21]. Together, these observations support the concept that asthmatics have an augmented risk of morbidity and mortality related to pH1N1 infection, and that this can be abrogated through IL-4Rα-targeted immunotherapy.

To investigate the effect of IL-4Rα mAb intervention in the context of allergic sensitization and pH1N1 infection, we measured viral load and the innate antiviral response in a time-course manner. We found that HDM-sensitized mice demonstrated an elevated viral load on day 8 post-infection and this was associated with a downregulation in IFN-β and ISGs shortly after pH1N1 inoculation, on day 4 post-infection. These data suggest that a dampened induction of the host’s antiviral response leads to elevated viral loads as a contributor to increased morbidity observed in HDM-sensitized mice.

While previous murine studies have shown rapid weight loss in mice following pH1N1 infection, to the best of our knowledge, it has yet to be determined how allergic sensitization affects morbidity and mortality [26]. House dust mite (*Dermatophagoides pteronyssinus*), an aeroallergen present in many households, is known to induce an allergic reaction in 85% of atopic patients [27]. HDM sensitivity has been reported in up to 130 million people worldwide, and studies have identified HDM as an environmental trigger and a predictor for the development of persistent asthma [27–29]. Der p 1, the major protease constituent in HDM, can initiate an early pro-inflammatory response by inducing the release of IL-6 and IL-8 in the airways [30]. Furthermore, HDM-induced inflammation in murine models results in disease and pathological phenotypes similar to those observed in human asthma. For example, HDM sensitization can induce GCM by promoting epithelial-mesenchymal transition, as well as eosinophilia through the induction of eotaxin release [31]. Using repeated intranasal HDM instillations to create an allergic phenotype, we were able to closely mimic the human clinical findings from the 2009 H1N1 pandemic in a murine model of allergic asthma, and showed that HDM-sensitized mice experienced significantly greater morbidity (as measured by weight loss) following pH1N1 infection [4, 5].

Several studies have indicated that bronchial epithelial cells from asthmatic patients are defective in the production of the antiviral cytokine, IFN-β, and as a result, allow rapid viral replication in vitro [32–34]. Our results from the time-course study indicate that a dampened induction of IFN-β occurs early in HDM-sensitized mice, starting on day 4 post-infection. An attenuated IFN-β response limits the expression of key ISGs required for viral clearance, including MX1 and IFITM3, which prevent viral entry; OAS1 and ISG-15, which inhibit viral translation and replication; viperin, which inhibits viral budding and release; and RIG-I, which is a pattern recognition receptor that induces the transcription of IFN-β [34, 35]. This dampened induction of IFN-β and downstream ISGs likely permits rapid viral replication in vivo, leading to elevated viral loads observed on day 8 post-infection, which was accompanied by elevated concentrations of the pro-inflammatory cytokines, IL-6 and IP-10.

While T_h_2 immune responses drive asthma phenotypes including eosinophilia and mucous hyperplasia, there is evidence to suggest it may also increase susceptibility to viral exacerbations [13, 14]. For example, in an experimental rhinovirus infection model, lung function impairment and elevated viral loads in asthmatics subjects were associated with an augmented IL-4, IL-5, and IL-13 response [16]. Murine asthma models have also shown that elevated IL-13 and dampened IFN-γ levels are associated with elevated viral loads and lung tissue destruction following pH1N1 infection [35]. Our data indicate that HDM-sensitized mice exhibit elevated IL-4 and IL-5 levels at various time points throughout pH1N1 infection, and conversely, lower IFN-γ on day 6 post-infection.

Due to its ability to relay signals for both IL-4 and IL-13, the blockade of IL-4Rα signalling has been of interest for alleviating asthma symptoms and severity. Murine asthma models have demonstrated a significant reduction in airway goblet cell metaplasia and eosinophilia following IL-4Rα mAb blockade [19]. Clinical trials using a human mAb targeting IL-4Rα have been successful in improving lung function and reducing peripheral blood eotaxin levels in moderate to severe eosinophilic asthma [20]. However, the role of IL-4Rα blockade has yet to be investigated in reducing morbidity resulting from highly virulent influenza A infection. Using our model of HDM-sensitization and pH1N1 infection, mice were subjected to a mAb targeting murine IL**-**4Rα through a prophylactic as well as a therapeutic treatment strategy. Our findings indicate that prophylactic treatment with IL-4Rα mAb not only reduced asthma phenotypes such as airway goblet cell metaplasia and eosinophilia, but also improved disease severity by reducing weight loss and viral load. In the therapeutic regimen, blockade of IL-4Rα attenuated weight loss and reduced viral load though eosinophilia and goblet cell metaplasia persisted. The exact mechanism for this observation is not known. One possibility is that the therapeutic dose of the monoclonal antibody was insufficient to completely block eosinophilia in tissue. Another possibility is that the therapeutic dose was not given sufficiently long enough to reverse these histological changes. Interestingly, with the therapeutic regimen, we did observe a significant reduction in eotaxin levels, an earlier marker of eosinophil recruitment, demonstrating the efficacy of IL-4Rα blockade.

## Conclusion

Our data indicate that allergic sensitization increases morbidity following pH1N1 infection by dampening anti-viral host defence pathways and increasing viral load and that prophylactic or therapeutic use of IL-4Rα blockade significantly improves disease expression and outcomes of mice sensitized to HDM. Together, these data suggest atopic asthmatics are at increased risk of severe influenza viral infections, which may be mitigated by therapeutic strategies involving IL-4Rα blockade.

## Supplementary Information

**Additional file 1: Table E1**. Primers used for qPCR assays.** Figure E1. **Lung Tissue Sectioning. The left lung was cut in a transverse manner to create a superior and an inferior segment. The inferior segment was embedded into paraffin, while the superior segment was further cut in a sagittal manner and embedded. **Figure E2.** House dust mite (HDM) sensitization delays viral clearance. Phosphate-buffered saline (PBS)-exposed or HDM-sensitized mice were infected with pandemic H1N1 (pH1N1) and sacrificed on days 0, 2, 4, 6, and 8 post-infection. (A) IFN-β protein expression was determined using ELISA on the supernatant of bronchoalveolar lavage fluid (BALF). (B) qPCR was performed on RNA from homogenized lung tissue to determine the number of viral RNA copies, and results were compared using a two-tailed Student’s t-test. Data (n=5 per group per day) were compared using a two-way ANOVA with Sidak’s multiple comparison’s test and are expressed as mean ± SEM. *P<0.05 after correction for multiple comparisons; ns: not significant. **Figure E3.** House dust mite (HDM) sensitization dampens the induction of interferon stimulated genes (ISGs). Phosphate-buffered saline (PBS)-exposed or HDM-sensitized mice were infected with pandemic H1N1 (pH1N1) and sacrificed on days 0, 2, 4, 6, and 8 post-infection. qPCR was performed on RNA from homogenized lung tissue to measure the gene expression of the interferon stimulated genes (A) MX1, (B) IFITM3, (C) OAS1, (D) ISG15, (E) Viperin, and (F) RIG-I. Results were normalized to expression on day 0 and were compared using a Mann-Whitney U test. Data (n=5 per group per day) are expressed as mean ± SEM. *P<0.05 after correction for multiple comparisons; **p<0.01 after correction for multiple comparisons; ns: not significant. **Figure E4.** House dust mite (HDM)-sensitized mice present elevated IL-33 and lower IFN-γ levels following pandemic H1N1 (pH1N1) infection. Phosphate-buffered saline (PBS)-exposed or HDM-sensitized mice were infected with pandemic H1N1 (pH1N1) and sacrificed through a time-course study. (A) IL-33 and (B) IFN-γ protein levels were measured using ELISA and multiplex assay, respectively (n=5 per group per day). Data were compared using two-way ANOVA with Sidak’s multiple comparison’s test and are expressed as mean ± SEM. *P<0.05 after correction for multiple comparisons; ** P<0.01 after correction for multiple comparisons; ns: not significant. **Figure E5.** Bronchoalveolar lavage fluid (BALF) cell differential analysis. BALF was obtained on day 8 post-infection from pandemic H1N1 (pH1N1)-infected mice. The percentage of (A-C) neutrophils, (D-F) lymphocytes, and (G-I) macrophages was determined in a total of 200 counted cells. (J-L) Trypan blue was used on the re-suspended BALF pellet and the absolute number of inflammatory cells in BALF was determined using a hemocytometer. Data were compared using a two-tailed Student’s t-test and are expressed as mean ± SEM. Blue/pink: intranasal phosphate-buffered saline (PBS) (n=10) / intranasal house dust mite extract (HDM) (n=9); green/orange: intranasal HDM followed by prophylactic strategy of IL-4Rα blockade (n=12) / IgG (n=12); black/red: intranasal HDM followed by therapeutic strategy of IL-4Rα blockade (n=12) / IgG (n=10). ** P<0.01; ***P<0.001; ns: not significant. **Figure E6.** Systemic blockade of Interleukin-4 receptor alpha (IL-4Rα) reduces IL-6 and IP-10 levels in bronchoalveolar lavage fluid (BALF). BALF was obtained on day 8 post-infection from pandemic H1N1 (pH1N1)-infected mice. (A-C) IL-6 and (D-F) IP-10 protein levels were measured in BALF by multiplex assay. Data were compared using the Mann-Whitney U test and are expressed as mean ± SEM. Blue/pink: intranasal phosphate-buffered saline (PBS) (n=10) / intranasal house dust mite extract (HDM) (n=9); green/orange: intranasal HDM followed by prophylactic strategy of IL-4Rα blockade (n=13) / IgG (n=12); black/red: intranasal HDM followed by therapeutic strategy of IL-4Rα blockade (n=12) / IgG (n=12). * P<0.05; ** P<0.01; ns: not significant. **Figure E7.** HDM-sensitization Induces Immune Cell Infiltration to the Airways. (A) PBS-exposed or HDM-sensitized mice were sacrificed on day 8 following pH1N1 infection or CAF exposure (n=10 except for HDM + pH1N1 n=9). Paraffin-embedded lung tissue obtained from these mice was sectioned and stained with Periodic Acid Schiff (PAS). Fractional lymphoid area was quantified and is expressed as a percentage of lymphoid tissue area per total cross sectional area. Data are expressed as mean ± SEM by one-way ANOVA. (B) PBS-exposed or HDM-sensitized nice were sacrificed through a time-course study (n=5 per group per day). The lungs were homogenized with PBS, and the supernatant was collected for protein analysis using the BCA assay). Data are expressed as mean ± SEM by a two-way ANOVA with Sidak’s multiple comparisons test. ** P<0.01; *** P<0.001.

## Data Availability

The primary data in this paper are available upon request to the senior author (D.D.S).

## Abbreviations

ANOVA: Analysis of variance
BALF: Bronchoalveolar lavage fluid
BM: Basement membrane
CAF: Chorioallantoic fluid
Ct: Cycle threshold
FFPE: Formalin fixed paraffin-embedded
GCM: Goblet cell metaplasia
HDM: House dust mite
IgG: Immunoglobulin G
IL: Interleukin
IL-4Rα: Interleukin-4 receptor-alpha
INF-β: Interferon-β
IP-10: Interferon-gamma induced protein 10
ISG: Interferon stimulated gene
mAB: Monoclonal antibody
mL: Millilitre
PAS: Periodic acid-Schiff
PBS: Phosphate-buffered saline
pH1N1: Influenza A virus pandemic strain subtype H1N1
qPCR: Quantitative polymerase chain reaction
RNA: Ribonucleic acid
SEM: Standard error of the mean
T_h_1: T helper type 1 cellular response
T_h_2: T helper type 2 cellular response
