# Supplementary material for: IL-4Rα blockade reduces influenza-associated morbidity in a murine model of allergic asthma
Source: Respir Res. 2021 Mar 2;22:75. doi: 10.1186/s12931-021-01669-0 (PMC7922715; doi:10.1186/s12931-021-01669-0)
Supplement: Supplementary file 1 — Additional file 1: Table E1. Primers used for qPCR assays. Figure E1. Lung Tissue Sectioning. The left lung was cut in a transverse manner to create a superior and an inferior segment. The inferior segment was embedded into paraffin, while the superior segment was further cut in a sagittal manner and embedded. Figure E2. House dust mite (HDM) sensitization delays viral clearance. Phosphate-buffered saline (PBS)-exposed or HDM-sensitized mice were infected with pandemic H1N1 (pH1N1) and sacrificed on days 0, 2, 4, 6, and 8 post-infection. (A) IFN-β protein expression was determined using ELISA on the supernatant of bronchoalveolar lavage fluid (BALF). (B) qPCR was performed on RNA from homogenized lung tissue to determine the number of viral RNA copies, and results were compared using a two-tailed Student’s t-test. Data (n=5 per group per day) were compared using a two-way ANOVA with Sidak’s multiple comparison’s test and are expressed as mean ± SEM. *P<0.05 after correction for multiple comparisons; ns: not significant. Figure E3. House dust mite (HDM) sensitization dampens the induction of interferon stimulated genes (ISGs). Phosphate-buffered saline (PBS)-exposed or HDM-sensitized mice were infected with pandemic H1N1 (pH1N1) and sacrificed on days 0, 2, 4, 6, and 8 post-infection. qPCR was performed on RNA from homogenized lung tissue to measure the gene expression of the interferon stimulated genes (A) MX1, (B) IFITM3, (C) OAS1, (D) ISG15, (E) Viperin, and (F) RIG-I. Results were normalized to expression on day 0 and were compared using a Mann-Whitney U test. Data (n=5 per group per day) are expressed as mean ± SEM. *P<0.05 after correction for multiple comparisons; **p<0.01 after correction for multiple comparisons; ns: not significant. Figure E4. House dust mite (HDM)-sensitized mice present elevated IL-33 and lower IFN-γ levels following pandemic H1N1 (pH1N1) infection. Phosphate-buffered saline (PBS)-exposed or HDM-sensitized mice were infected with pandemic [file 12931_2021_1669_MOESM1_ESM.docx]

**Additional files**

**METHODS**

**Acute House Dust Mite Sensitization and Pandemic H1N1 Infection Model**

HDM (*Dermatophagoides pteronyssinus*) (Greer, Lenoir, NC) was prepared by dissolving 25 µg of protein antigen in 35 µL of PBS. Mice were intranasally exposed to either HDM or PBS, 5 days per week for two consecutive weeks. This was followed by a single intranasal 50 µL dose of pH1N1 (10^6.4^ EID_50_/mL) on day 0. The pH1N1 influenza A virus strain (A/California/04/2009) was obtained from BEI resources and amplified in embryonated chicken eggs. Control animals received 50 µL of CAF from non-infected eggs instead. HDM/PBS instillations then followed on days 2 to 6 (Figure E1A). Mice were weighed daily and were sacrificed on day 8 to perform outcome measurements (n=10 per group, except n=9 for HDM + pH1N1). Two time-course studies were performed using the same protocol to interrogate disease progression; mice were sacrificed either on days 0, 2, 4, 6, and 8 post-infection, or on days 0, 1, 3, 5, and 8 post-infection (n=5 per group per day).

**Prophylactic and Therapeutic Intervention Designs**

HDM-sensitized and pH1N1-infected mice were subjected to either a prophylactic or a therapeutic strategy of IL-4Rα blockade (AMG 317). 2 mg of anti-IL-4Rα (4-3) (n=13) or IgG1 isotype control (4G8) (n=12) monoclonal antibodies (Amgen, Seattle, WA) were administered intraperitoneally 2, 7, and 12 days prior to and 3 days following pH1N1 infection to create a prophylactic design. In contrast, anti-IL-4Rα or IgG1 isotype control were administered on days 1 and 6 following pH1N1 infection to create a therapeutic design (n=12 per group) (Figure E1B). Control animals were intranasally exposed to PBS or HDM, and received intraperitoneal administrations of PBS, the vehicle control. Mice were weighed daily and were sacrificed on day 8 post-infection to perform outcome measurements.

**BALF, Plasma, and Lung Tissue Processing**

BALF was collected from the right lung using 600 µL of PBS and was centrifuged at 300 x g for 10 minutes. Total cell counts were performed on the re-suspended pellet using a hemocytometer. Cytocentrifuge slides were prepared and stained with Wright-Giemsa (Sigma Aldrich, MO, USA) to perform BALF differentials. Blood was collected from the inferior vena cava into ethylenediaminetetraacetic acid (EDTA)-coated tubes and was centrifuged at 2000 x g for 10 minutes to separate plasma. Samples were then stored at -80^o^C for future analysis.

The right lung was flash frozen and stored at -80^o^C. Lung homogenization was later performed by adding 500 µL of PBS and a single 7mm DNAse/RNAse free bead. Samples were then shaken at 50 Hz for 4 minutes using the TissueLyser LT system (QIAGEN, CA, USA). 25 µL from the homogenized lung tissue was stored separately for viral titre quantification, while the rest was centrifuged at 10,000 x g for 10 minutes. The supernatant was separated and stored at -80^o^C for future protein analysis using ELISA and the BCA assay.

**qPCR**

RNA extraction was performed on homogenized lung tissue using RNeasy Plus Mini Kit (QIAGEN, CA, USA) per manufacturer’s protocol. To quantify viral load, viral RNA was extracted from an avian egg-amplified virus stock using a QIAamp viral RNA kit (QIAGEN, CA, USA), diluted to generate a range of standards containing between 10^3^ to 10^9^ RNA copies, and mixed with 50 ng of cellular RNA from virus-free murine lungs. qPCR was performed on triplicates of RNA samples and viral standards using ROX™ qScript™ One-Step Fast qRT-PCR Kit (Quanta Biosciences, MD, USA) and 50 ng total RNA per reaction. The primers and probe used were based on the highly conserved Influenza A matrix gene, and are as follows: 5′-CTT CTA ACC GAG GTC GAA ACG-3′ (forward primer, FluA-M52C); 5′-AGG GCA TTT TGG ACA AAG/T CGT CTA-3′ (reverse primer, FluA-M253R); Fam-CCGTCAGGCCCCCTCAAAGC-BHQ1 (probe, FluA-M96_taq).

To quantify the expression of ISGs, cDNA was made from RNA samples using iScript™ cDNA synthesis kit (Bio-Rad, WA, USA). qPCR was performed using iTaq™ Universal SYBR® Green Supermix (Bio-Rad, WA, USA) on a CFX384 Touch™ Real-Time PCR Detection System (Bio-Rad, WA, USA), and the primers are listed in Table E1. Gene expression was quantified relative to the expression of the control gene GAPDH and analyzed using the comparative Ct method (ΔΔCt).

**Lung Tissue Sectioning and Morphometry**

Following fixation in 10% formalin, a transverse cut was made into the left lung to create a superior and an inferior segment. The inferior segment was embedded into paraffin, while the superior segment was further cut in a sagittal manner and embedded (Figure E2). Three-micron thick sections were stained with Periodic Acid-schiff (PAS), scanned with Aperio ScanScope AT2, and analyzed by applying a colour segmentation algorithm using Aperio ImageScope software (Leica Biosystems, Wetzlar, Germany). The airway epithelium and the airway wall were traced and quantified using Aperio ImageScope software and are represented relative to the basement membrane length.

**Cytokine/Chemokine Measurements**

A Mouse/Rat IL-33 Quantikine ELISA kit (R&D Systems, MN, USA) was used to measure IL-33 in lung homogenate supernatant, and a Mouse IFN Beta ELISA Kit, High Sensitivity (PBL Assay Science, NJ, USA) was used to measure IFN-β in BALF. IL-4, IL-5, and IFN-γ were measured in BALF using a Mouse High Sensitivity T Cell Panel, while eotaxin, IL-6, and IP-10 were measured in plasma and BALF using a Mouse Cytokine/Chemokine Magnetic Bead Panel (EMD Millipore, Darmstadt, Germany).

**FIGURES**

**Table E1 – Primers used for qPCR assays**

| **Gene** | **Forward (5'-3')** | **Reverse (5'-3')** |
| --- | --- | --- |
| OAS1 | ATTACCTCCTTCCCGACACC | CAAACTCCACCTCCTGATGC |
| RIG-I | CCACCTACATCCTCAGCTATATGA | TGGGCCCTTGTTGTTCTTCT |
| MX-1 | GAAGGCAAGGTCTTGGATG | GCTGACCTCTGCACTTGACT |
| IFITM3 | GGATTCCGACTTCCGGTCCT | GTGTTACACCTGCGTGTAGGG |
| ISG-15 | TGACGCAGACTGTAGACACG | CAGCCAGAACTGGTCTTCGT |
| Viperin | CTTCAACGTGGACGAAGACA | GACGCTCCAAGAATGTTTCA |
| GAPDH | ACCCAGAAGACTGTGGATGG | GGATGCAGGGATGATGTTCT |

**
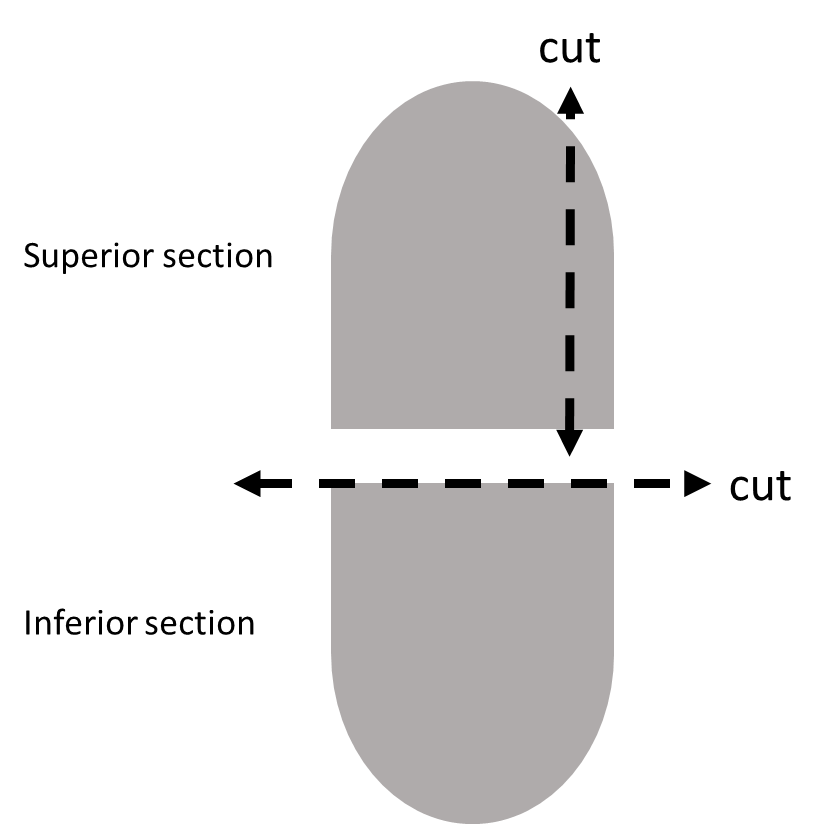
**

**Figure E1 – Lung Tissue Sectioning.** The left lung was cut in a transverse manner to create a superior and an inferior segment. The inferior segment was embedded into paraffin, while the superior segment was further cut in a sagittal manner and embedded.

**Figure E2 - House dust mite (HDM) sensitization delays viral clearance.** Phosphate-buffered saline (PBS)-exposed or HDM-sensitized mice were infected with pandemic H1N1 (pH1N1) and sacrificed on days 0, 2, 4, 6, and 8 post-infection. (A) IFN-β protein expression was determined using ELISA on the supernatant of bronchoalveolar lavage fluid (BALF). (B) qPCR was performed on RNA from homogenized lung tissue to determine the number of viral RNA copies, and results were compared using a two-tailed Student’s t-test. Data (n=5 per group per day) were compared using a two-way ANOVA with Sidak’s multiple comparison’s test and are expressed as mean ± SEM. *P<0.05 after correction for multiple comparisons; ns: not significant.

**Figure E3 - House dust mite (HDM) sensitization dampens the induction of interferon stimulated genes (ISGs).** Phosphate-buffered saline (PBS)-exposed or HDM-sensitized mice were infected with pandemic H1N1 (pH1N1) and sacrificed on days 0, 2, 4, 6, and 8 post-infection. qPCR was performed on RNA from homogenized lung tissue to measure the gene expression of the interferon stimulated genes (A) MX1, (B) IFITM3, (C) OAS1, (D) ISG15, (E) Viperin, and (F) RIG-I. Results were normalized to expression on day 0 and were compared using a Mann-Whitney U test. Data (n=5 per group per day) are expressed as mean ± SEM. *P<0.05 after correction for multiple comparisons; **p<0.01 after correction for multiple comparisons; ns: not significant.

**Figure E4 – House dust mite (HDM)-sensitized mice present elevated IL-33 and lower IFN-γ levels following pandemic H1N1 (pH1N1) infection.** Phosphate-buffered saline (PBS)-exposed or HDM-sensitized mice were infected with pandemic H1N1 (pH1N1) and sacrificed through a time-course study. (A) IL-33 and (B) IFN-γ protein levels were measured using ELISA and multiplex assay, respectively (n=5 per group per day). Data were compared using two-way ANOVA with Sidak’s multiple comparison’s test and are expressed as mean ± SEM. *P<0.05 after correction for multiple comparisons; ** P<0.01 after correction for multiple comparisons; ns: not significant.

**Figure E5 – Bronchoalveolar lavage fluid (BALF) cell differential analysis.** BALF was obtained on day 8 post-infection from pandemic H1N1 (pH1N1)-infected mice. The percentage of (A-C) neutrophils, (D-F) lymphocytes, and (G-I) macrophages was determined in a total of 200 counted cells. (J-L) Trypan blue was used on the re-suspended BALF pellet and the absolute number of inflammatory cells in BALF was determined using a hemocytometer. Data were compared using a two-tailed Student’s t-test and are expressed as mean ± SEM. Blue/pink: intranasal phosphate-buffered saline (PBS) (n=10) / intranasal house dust mite extract (HDM) (n=9); green/orange: intranasal HDM followed by prophylactic strategy of IL-4Rα blockade (n=12) / IgG (n=12); black/red: intranasal HDM followed by therapeutic strategy of IL-4Rα blockade (n=12) / IgG (n=10). ** P<0.01; ***P<0.001; ns: not significant.

**
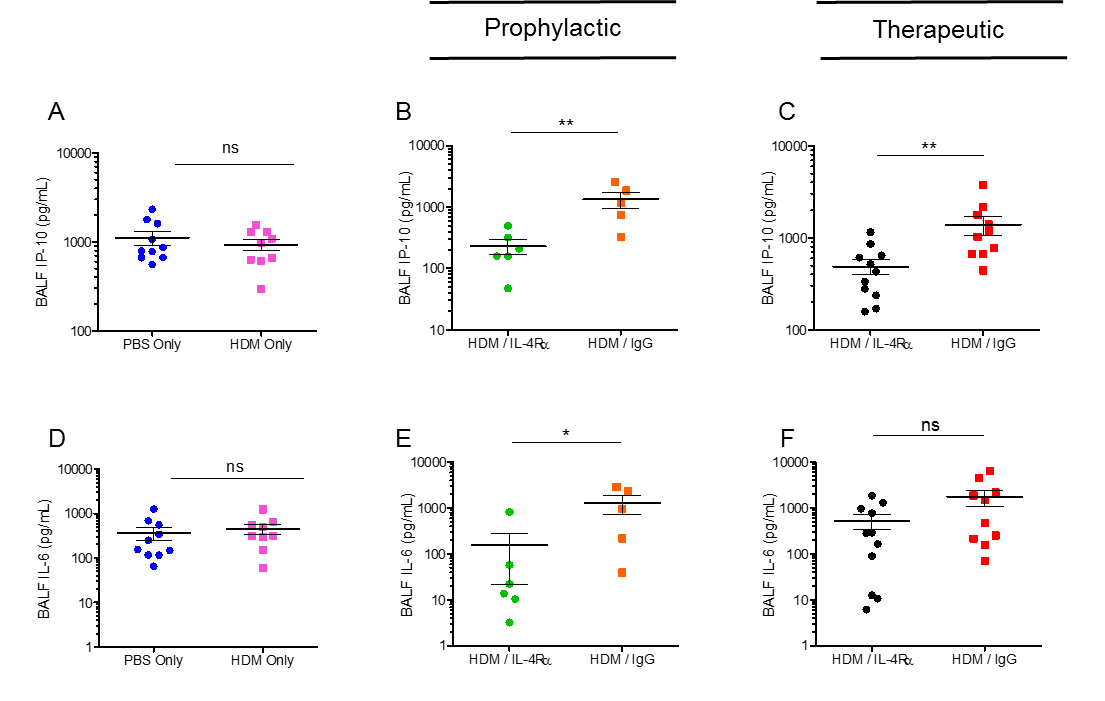
**

**Figure E6 - Systemic blockade of Interleukin-4 receptor alpha (IL-4Rα) reduces IL-6 and IP-10 levels in bronchoalveolar lavage fluid (BALF).** BALF was obtained on day 8 post-infection from pandemic H1N1 (pH1N1)-infected mice. (A-C) IL-6 and (D-F) IP-10 protein levels were measured in BALF by multiplex assay. Data were compared using the Mann-Whitney U test and are expressed as mean ± SEM. Blue/pink: intranasal phosphate-buffered saline (PBS) (n=10) / intranasal house dust mite extract (HDM) (n=9); green/orange: intranasal HDM followed by prophylactic strategy of IL-4Rα blockade (n=13) / IgG (n=12); black/red: intranasal HDM followed by therapeutic strategy of IL-4Rα blockade (n=12) / IgG (n=12). * P<0.05; ** P<0.01; ns: not significant.

**Figure E7 – HDM-sensitization Induces Immune Cell Infiltration to the Airways.** (A) PBS-exposed or HDM-sensitized mice were sacrificed on day 8 following pH1N1 infection or CAF exposure (n=10 except for HDM + pH1N1 n=9). Paraffin-embedded lung tissue obtained from these mice was sectioned and stained with Periodic Acid Schiff (PAS). Fractional lymphoid area was quantified and is expressed as a percentage of lymphoid tissue area per total cross sectional area. Data are expressed as mean ± SEM by one-way ANOVA. (B) PBS-exposed or HDM-sensitized nice were sacrificed through a time-course study (n=5 per group per day). The lungs were homogenized with PBS, and the supernatant was collected for protein analysis using the BCA assay). Data are expressed as mean ± SEM by a two-way ANOVA with Sidak’s multiple comparisons test. ** P<0.01; *** P<0.001

**Figure E8 – HDM sensitization Increases Epithelial and Airway Wall Thickness.** PBS-exposed or HDM-sensitized mice were sacrificed on day 8 following pH1N1 infection or CAF exposure (n=10 except for HDM + pH1N1 n=9). (A) The airway epithelium and (B) the airway wall were traced and quantified and are represented relative to the basement membrane length.
